# Continuous Taurocholic Acid Exposure Promotes Esophageal Squamous Cell Carcinoma Progression Due to Reduced Cell Loss Resulting from Enhanced Vascular Development

**DOI:** 10.1371/journal.pone.0088831

**Published:** 2014-02-14

**Authors:** Sho Sato, Hiroto Yamamoto, Ken-ichi Mukaisho, Shota Saito, Takanori Hattori, Gaku Yamamoto, Hiroyuki Sugihara

**Affiliations:** 1 Department of Pathology, Division of Molecular and Diagnostic Pathology, Shiga University of Medical Science, Shiga, Japan; 2 Department of Oral and Maxillofacial Surgery, Shiga University of Medical Science, Shiga, Japan; Ottawa Hospital Research Institute, Canada

## Abstract

**Background:**

Refluxogenic effects of smoking and alcohol abuse may be related to the risk of esophageal squamous cell carcinoma (ESCC). The present study attempts to clarify the effects of continuous taurocholic acid (TCA) exposure, which is neither mutagenic nor genotoxic, on ESCC progression.

**Methods:**

A squamous carcinoma cell line (ESCC-DR) was established from a tumor induced in a rat model of gastroduodenal reflux. ESCC-DR cells were incubated with 2 mM TCA for ≥2 months. The effects of continuous TCA exposure were evaluated *in vitro* on cell morphology, growth, and invasion and *in vivo* on xenograft tumor growth in nude mice. Moreover, the mean level of secreted transforming growth factor (TGF)-β1 and vascular endothelial growth factor (VEGF) proteins in cell culture supernatants and mRNA synthesis of TGF-β1 and VEGF-A of ESCC cells were measured. The angiogenic potential was further examined by a migration assay using human umbilical vein endothelial cells (HUVECs).

**Results:**

Continuous TCA exposure induced marked formation of filopodia *in vitro*. Expression levels of angiogenic factors were significantly higher in the cells treated with TCA than in control cells. Tumor xenografts derived from cells pre-exposed to TCA were larger and more vascularized than those derived from control cells. In addition, TCA exposure increased HUVEC migration.

**Conclusion:**

Continuous TCA exposure enhanced ESCC progression due to reduced cell loss *in vivo*. Cell loss was inhibited by TCA-induced vascular endothelial cell migration, which was mediated by TGF-β1 and VEGF-A released from ESCC cells.

## Introduction

Esophageal cancer can arise as esophageal squamous cell carcinoma (ESCC) or esophageal adenocarcinoma (EAC), which have distinct etiological and pathological characteristics. Many patients with esophageal cancer develop ESCC in Asian countries [1]. It is widely accepted that ESCC is associated with smoking and alcohol consumption [2], [3]. Tobacco and alcohol abuse decrease lower esophageal motility, delay gastric emptying, and increase gastric secretion [4]–[8]. The refluxogenic effects of smoking and alcohol abuse induce gastroduodenal reflux, which is associated with the development of ESCC. Gastrectomy is also associated with the subsequent development of distal esophageal cancer [9]–[11], and symptomatic duodenogastric reflux is the most common post-gastrectomy syndrome [12], [13]. Moreover, several research groups including ours have demonstrated that not only EAC but also ESCC can develop in animal models of gastroduodenal reflux with bile acids [14]–[18].

Bile acids are one of the most toxic factors for mucosal injury in the carcinogenesis of the upper digestive tract [19], [20]. Although there is a wide variation in the relative individual toxicity of bile acid fractions, various mechanisms of bile acids effects on cancer development and progression have been reported. Jürgens et al. reported that Barrett’s carcinoma cell lines and patients’ metaplastic Barrett’s esophagus tissues are resistant to deoxycholic acid (DCA)-induced apoptosis (despite the induction of DNA damage) because of the activation of the NF-κB cell survival pathway [19]. Song et al. showed that cyclooxygenase 2(Cox2) is involved in cancer progression elicited by DCA through Erk and Akt signaling [21]. A recent study also suggests that chenodeoxycholic acid (CDCA) stimulates the development of human esophageal cancer by promoting angiogenesis through the Cox2 pathway [22].

In the present study, we attempted to clarify the effects of continuous taurocholic acid (TCA) exposure at a high concentration (2 mM) on ESCC progression on the basis of the following findings: (i) most unconjugated bile acids and glycine conjugates whose p*K*a values are >4 precipitate in the gastric acidic environment, whereas taurine conjugates are soluble even at pH 2 [23], [24]; (ii) 23% of patients who underwent a partial gastrectomy experienced acid (pH <4) reflux into the distal esophagus [13]; (iii) Graffner *et al*. reported that the median total conjugated bile acid concentration was significantly higher in patients who underwent gastric resection (3236 µM) than in patients in the control group (349 µM), and the range of TCA concentration was 141 µM to >1000 µM [25]. Iftikar *et al.* also reported that two patients who underwent partial gastrectomy had total bile acids concentration of 14655 µM and 18620 µM [26].

The influence of tumor angiogenesis on cancer progression has been debated over the last decades. In the clinical setting, high rates of transforming growth factor (TGF)-β1, vascular endothelial growth factor (VEGF), and Cox2 expression have been found to be associated with poor prognosis in patients with esophageal cancer [27]–[30]. To explore the role of angiogenesis on cancer progression induced by continuous TCA exposure, we analyzed protein and mRNA expression levels of angiogenic factors. We demonstrate that continuous TCA exposure promotes ESCC progression through reduced cell loss induced by TGF-β1 and VEGF-mediated neovascularisation.

## Materials and Methods

### Cell Culture and TCA Treatment

We used ESCC-DR cells that were established from a tumor induced in a rat model of gastroduodenal reflux [17]. The cells were grown and maintained in Dulbecco’s modified Eagle’s medium (DMEM; Nacalai Tesque, Kyoto, Japan) supplemented with 1% antibiotic–antimycotic solution (Gibco, NY, USA) and 10% fetal bovine serum (FBS; PAA Laboratories, Pasching, Austria) in a humidified incubator containing 5% CO_2_ at 37°C [20]. The cells were incubated in the growth medium containing 2 mM taurocholic acid sodium salt hydrate (TCA, SIGMA, St. Louis, USA) for ≥2 months before analysis. These cells were termed “tca.” ESCC-DR cells cultured in the growth medium without TCA over the same period were used as a control in this study.

### Flow Cytometry for Cell Cycle Analysis

The cells seeded in 75-cm^2^ flasks were exposed to 2 mM TCA or 300 µM deoxycholic acid (DCA, Sigma) for 24 h. They were harvested, washed with PBS, and fixed with 70% ethanol at room temperature for 30 min. The fixed cells were centrifuged and washed with PBS thrice. They were then resuspended in 0.5 mL of PBS containing 2 mg/mL RNase A (Sigma) at 37°C for 30 min and stained with 50 µg/mL propidium iodide (Nacalai Tesque) at 4°C for 1 h. The cellular DNA content was measured using FACSCalibur (Becton Dickinson, NJ, USA).

### Cell Growth Assay

An MTT assay was used to evaluate cell growth. The control and tca cells were seeded in 12-well plates (1×10^4^ cells/well). After 3, 24, 48, 72, or 96 h of incubation, medium containing 0.25 mg/mL MTT was added to the calls (DOJINDO, Kumamoto, Japan). Formazan crystals were dissolved in DMSO, and absorbance was measured at 570 nm using an Infinite M200 microplate reader (TECAN, Männedorf, Switzerland).

### Preparation of Cell Lysate and Western Blotting

The following primary antibodies were used to perform western blotting: Akt (pan) mouse mAb (cat. #2920, Cell Signlaing, MA, USA), Phospho-Akt (Ser473)(D9E)XP rabbit mAb (cat. #4060, Cell Signaling), p44/42 MAP Kinase (L34F12) mouse mAb (cat. #4696, Cell Signaling), Phospho-p44/42 MAPK (Erk1/2) (Thr202/Tyr204) (D13.14.4E) XP Rabbit mAb (cat. #4370, Cell Signaling), Anti-Rat Cox2 Rabbit IgG Affinity Purify (cat. 18955, IBL, Gunma, Japan), and β-actin (C4) mouse mAb (cat. sc-47778, Santa Cruz, CA, USA). Goat peroxidase-conjugated anti-rabbit IgG (cat. ab6721, Abcam, Cambridge, UK) and goat peroxidase-conjugated anti-mouse IgG (cat. AP124P, Millipore, MA, USA) were used as secondary antibodies.

The cells were washed with PBS and lysed in lysis buffer [50 mM Tris–HCl, pH 7.4; 150 mM NaCl; 0.5 mM ethylenediaminetetraacetic acid (EDTA); 1% Nonidet P-40] containing a mixture of protease inhibitors (1 mM phenylmethylsulfonyl fluoride, 1 µg/mL leupeptin, 1 µg/mL pepstatin A, and 0.09 U/mL aprotinin) and 1% phosphate inhibitor cocktail II (Sigma). After incubation at 4°C for 30 min and mixing with a vortex mixer, the cell lysates were centrifuged at 12,000×*g* at 4°C for 10 min. The supernatants were collected, and the protein content was quantified using the BCA protein assay reagent (Thermo Fisher Scientific, Waltham, MA, USA).

PAGE was performed according to the manufacturer’s instructions (NuPAGE kit; Invitrogen, CA, USA). Protein samples were solubilized in NuPAGE LDS sample buffer and incubated at 70°C for 10 min after addition of 2% β-mercaptoethanol. Proteins were separated on a 4%–12% SDS-PAGE gradient gel (NuPAGE Bis-Tris Gel) and electrotransferred onto nitrocellulose membranes (Invitrogen). The nitrocellulose membranes were blocked with 4% nonfat dried milk in TBS-T buffer (10 mM Tris–HCl, pH 7.4; 150 mM NaCl; 0.1% Tween-20) and incubated with a primary antibody at 4°C overnight. After incubation with a horseradish peroxidase (HRP)-conjugated secondary antibody at room temperature for 1 h, protein bands were visualized with an HRP substrate (Millipore) and scanned on a luminescent imaging analyzer LAS-4000plus (Fuji Film, Tokyo, Japan).

### Quantitative Reverse Transcription-polymerase Chain Reaction (qRT-PCR)

Total RNA was isolated using the RNeasy kit (QIAGEN, Hilden, Germany); cDNA was synthesized from 2 µg of each total RNA sample. The cDNA samples were subjected to qRT-PCR (LightCycler 480, Roche, Basel, Switzerland) using the primers given below and SYBR Premix Ex Taq II (Takara Bio, Otsu, Japan). All PCR primers were purchased from Takara Bio. Their sequences were as follows: 5′-CATTGCTGTCCCGTGCAGA-3′ and 5′-AGGTAACGCCAGGAATTGTTGCTA-3′ for TGF-β1; 5′-GCACGTTGGCTCACTTCCAG-3′ and 5′-TGGTCGGAACCAGAATCTTTATCTC-3′ for VEGF-A;

5′-GGCACAGTCAAGGCTGAGAATG-3′ and 5′-ATGGTGGTGAAGACGCCAGTA-3′ for glyceraldehyde 3-phosphate dehydrogenase (GAPDH). PCR was performed using the following conditions: initial denaturation at 95°C for 30 s, then 40 cycles of 95°C for 5 s, and annealing and elongation at 60°C for 20 s. mRNA expression levels were normalized to the mRNA levels of the internal standard gene, GAPDH.

### Enzyme-linked Immunosorbent Assay (ELISA)

The control and tca cells were seeded in 12-well plates (1×10^4^ cells/well). After 24 h, the cell culture medium was collected and centrifuged for 5 min at 14,000×*g* (4°C) to remove the detached cells. The total TGF-β1 and VEGF levels in the supernatant were measured using ELISA kits (R&D Systems, Minneapolis, USA) according to the manufacturer’s instructions.

### Cell Migration Assay of Vein Endothelial Cells

Human umbilical vein endothelial cells (HUVEC-2) were purchased from BD Biosciences (California, USA) and grown in a basal medium (Medium 200; Gibco) containing supplements and serum (Low Serum Growth Supplement; Gibco). Migration assays were performed using the BD BioCoat Angiogenesis System for endothelial cell migration (354143, BD Biosciences). The control and tca cells were seeded in 12-well plates (1×10^4^ cells/well). After 24 h, cell culture medium was collected and centrifuged for 5 min at 14,000×*g* (4°C). The supernatant was added to the lower chamber of transwell plates under each well. HBSS (Sigma) was added to one of the lower chambers as a negative control (without supernatant of culture medium). HUVECs starved of serum for 5 h were harvested using trypsin–EDTA and resuspended in the basal medium. The suspensions were added to each top chamber at a density of 5×10^5^ cells/mL. Both chambers were incubated for 22 h at 37°C to induce migration through the fibronectin-coated membranes. The top chambers were then transferred to wells containing Calcein AM (BD Biosciences) in HBSS. These samples were incubated for 1.5 h at 37°C. The level of fluorescence was measured using a plate reader (Infinite M200; TECAN) at 494/517 nm. Data were calculated as relative ratios in comparison to an HBSS negative control. Immunofluorescence images were visualized using an Olympus BX-61 fluorescent microscope (Olympus, Tokyo, Japan), and images were captured with a CoolSNAP-HQ camera (NIPPON ROPER, Tokyo, Japan).

### Electron Microscopy

The morphology of control and tca cells was analyzed using electron microscopy. The cells were fixed in glutaraldehyde and embedded in epoxy resin. Thin sections were double-stained with lead citrate and uranyl acetate and examined under a JEOL JEM-1200EX transmission electron microscope (Japan Electron Optics Laboratory, Tokyo, Japan).

### Invasion Assay of Cancer Cells

This assay was performed using BD BioCoat Matrigel invasion chambers with 8-µm pore inserts (354165, BD Biosciences). The cells suspended in serum-free DMEM were seeded in the top chambers of the transwell plate at a density of 5×10^4^ cells/mL, whereas DMEM containing 5% FBS was added to the lower chambers as a chemoattractant. Both chambers were incubated for 48 h at 37°C to induce invasion through the Matrigel-coated membranes. The upper chambers were then transferred to wells containing Calcein AM (BD Biosciences) in HBSS (Gibco), as described above, and incubated for 1 h at 37°C; the signals were quantified on a fluorescence plate reader (Infinite M200; TECAN) at 485/530 nm. The invasiveness of tca cells was calculated as a fluorescence intensity ratio relative to that of control cells.

### Xenograft Tumor Growth Assay

All procedures were in compliance with the Ethical Guidelines for Animal Experimentation and Care and Use of Laboratory Animals at Shiga University of Medical Science, Japan. The protocol was approved by the Committee on the Ethics of Research Center for Animal Life Science at Shiga University of Medical Science (Permit number: 2011-12-2). All efforts were made to minimize animal suffering and reduce the number of animals used. Five-week-old athymic BALB/cA nu/nu nude mice (CLEA Japan, Tokyo, Japan) were subcutaneously inoculated with 1×10^6^ tca or control cells. Tumor length and width were measured every 4 days, and tumor volume (TV) was estimated using the following formula: TV (mm^3^) = (width^2^×length)/2. The animals were sacrificed with an overdose of isoflurane 4 weeks after the inoculation. Resected tumors that developed on the back of the mice were fixed with 10% formalin (in PBS) for 4 h and embedded in paraffin. Serial 3-µm sections were used for histological evaluation by hematoxylin–eosin (HE) staining, CD31 and Ki-67 immunohistochemical stainings, and apoptosis detection.

### Evaluation of Tumor Necrosis in Tumor Xenografts

The necrotic proportion of each tumor xenografts was assessed using HE staining. The area of tumor necrosis was measured using a Leica LMD6000 system (Leica, Wetzlar, Germany).

### Apoptosis Detection

Apoptotic cells were detected using an *in situ* apoptosis detection kit (Takara Bio). The staining procedures were performed according to the manufacturer’s instructions. After deparaffinization, the tissue was briefly digested with proteinase K (20 µg/mL in PBS) for 15 min at room temperature and washed with PBS. The slides were then incubated in 3% hydrogen peroxide for 5 min, followed by another wash with PBS. The slides were incubated with TdT enzyme and substrate at 37°C for 90 min, washed and then incubated with an HRP-conjugated anti-FITC antibody 30 min. Finally, they were washed, stained with diaminobenzene (Nichirei, Tokyo, Japan), and counterstained with hematoxylin.

### Immunohistochemical Detection of CD31 and Ki-67

Immunohistochemical staining was performed using an anti-CD31 rabbit polyclonal antibody (cat. #ab28364; Abcam) and an anti-Ki-67 rabbit monoclonal antibody (cat. # ab16667; Abcam). The staining was performed using a modified streptavidin–peroxidase conjugate method based on the poly-HRP anti-mouse/rabbit IgG detection system [Polymer Detection System, Histofine MAX-PO (MULTI); Nichirei, Tokyo, Japan].

### Quantification of Blood Vessel Density in Tumor Xenografts

The number of blood vessels positive for CD31 was manually counted in the entire tumor area, which was determined using the Leica LMD6000 system in the available sections.

### Calculation of Ki-67 and Apoptosis Indices

The percentages of Ki-67-positive and apoptotic tumor cells (separately) were determined in 5 randomly selected visual fields (magnification×400).

### Statistical Analysis

Each experiment was performed in triplicate. All data were calculated as mean ± SD. All calculations were performed in Microsoft Excel. Comparison between the groups was performed using Student’s *t*-test. The differences were considered statistically significant at *P*<0.05.

## Results

### Effects of TCA Treatment on Cell Growth *in vitro*

#### Cell cycle

**Figure 1:**
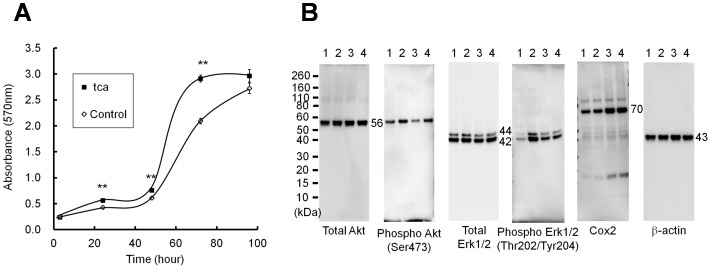
Effects of TCA exposure on ESCC-DR cell growth and signaling. Cell growth rates were evaluated using the MTT assay (A) Values represent the mean ± S.D. of three independent experiments. TCA exposure promoted the growth of ESCC-DR cells compared with the control treatment (**P<0.01). (B) Nos.1 and 2 are control cells and Nos.3 and 4 are tca cells. Nos.1 and 3 were treated without TCA. Nos.2 and 4 were treated with 2 mM TCA for 48 h. Ten micrograms of the whole cell lysate were subjected to western blotting with antibodies against total Akt, pAkt, total Erk1/2, pErk1/2, Cox 2, and β-actin. β-actin was used as an internal control. Molecular weights of Akt, Erk1/2, Cox2, and β-actin are indicated in the figures.

We examined the effects of TCA treatment on cell cycle using FACS analysis (Fig. 1A). 24-h incubation with 300 µM DCA induced a G1 arrest and a small amount of apoptosis in control cells. In contrast, 24 h of incubation with 2 mM TCA enhanced the entry into the S-phase in both the control and tca cells. There was no significant difference between the control and tca cells cultured without bile acids (Table 1).

**Table 1:** Effects of TCA treatment on cell cycle using FACS analysis.

Control	Sub G1	G1/G0	S	G2/M
Without bile acids	0.20±0.25	48.64±0.42	40.47±0.08	10.89±0.43
TCA 2 mM	0.15±0.05	39.79±0.16*	48.86±0.37*	11.35±0.52
DCA 300 µM	2.08±0.22*	74.58±0.62*	18.16±0.52*	7.26±0.28*
**tca**	**Sub G1**	**G1/G0**	**S**	**G2/M**
Without bile acids	0.14±0.06	48.35±0.59	38.98±0.59	12.68±0.15
TCA 2 mM	0.09±0.06	42.75±0.31*	45.39±0.16*	11.87±0.34

*p<0.01.

Cells were cultured in the medium without bile acids until cell density had become near 40% of confluence. Then cells were treated with or without bile acids for 24-h. The cellular DNA content was evaluated by flow cytometry. Statistical analysis was performed using Student’s t-test to compare the cell number between with and without bile acids in each cell cycle.

#### Cell growth

Cell growth rate was assessed using the MTT assay (Fig. 1A). Tca cells treated with 2 mM TCA showed increased growth rate compared with control cells without TCA [MTT absorbance values after 72 h: tca, 2.92±0.06 (mean ± SD); control, 2.10±0.05; *P* = 0.0001]. These results showed that TCA exposure increased cell growth by accelerating the entry into the S phase.

#### Signaling pathway

The expression of total Akt, Phospho-Akt, total Erk, Phospho-Erk, and Cox2 are shown in Figure 1B.

According to cell growth results, Akt phosphorylation was transiently induced by TCA in the control and tca cells. Erk phosphorylation was induced by TCA in control cells. In tca cells, TCA did not induce Erk phosphorylation, but the baseline phosphorylation was higher than that of control cells. Both Akt and Erk were activated by TCA in control cells, but Cox2 expression was not induced. Although TCA could not significantly enhance Cox2 expression, the expression level of Cox2 in tca cells was higher than that in control cells.

### Effects of TCA Treatment on Angiogenic Factors *in vitro*

#### TGF-β1 and VEGF-A gene expression

**Figure 2:**
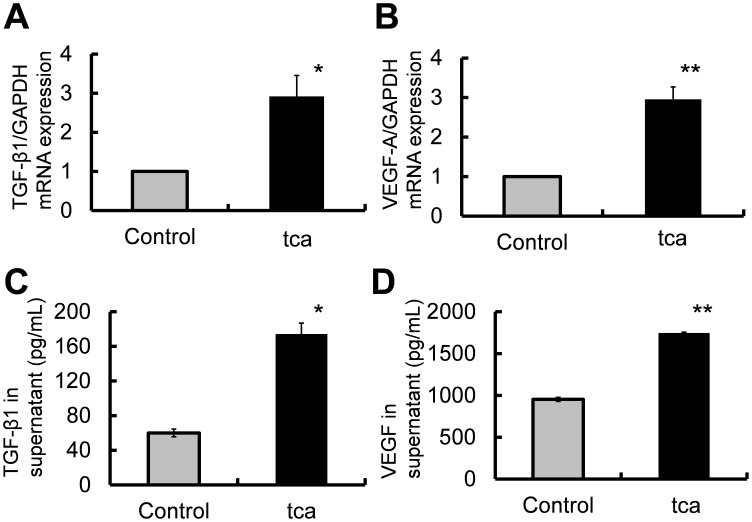
Quantification of TGF-β1 and VEGF-A mRNA and protein levels. (A, B) Control cells were cultured in the medium without TCA and tca cells were cultured in the medium with 2 mM TCA. Total RNA was extracted from these cells and expression levels of TGF-β1 and VEGF-A mRNA were evaluated by qRT-PCR. (C, D). Culture supernatants were collected from control cells incubated in the medium without TCA and tca cells incubated in the medium with 2 mM TCA for 24 h. (A) TGF-β1 mRNA levels in tca and control cells (*P<0.05). (B) VEGF-A mRNA levels in tca and control cells (**P<0.01). (C) Comparison of the mean level of secreted TGF-β1 in the media from tca vs. control cultures (**P<0.01). (D) Comparison of the mean level of secreted VEGF protein for tca vs. control cells (**P<0.01).

TGF-β1 and VEGF-A mRNA levels were quantified using qRT-PCR. TGF-β1 mRNA expression in tca cells was 2.92±0.53-fold higher than that in control cells (*P* = 0.0266; Fig. 2A). VEGF-A mRNA expression in tca cells was 2.96±0.31-fold higher than that in control cells (*P* = 0.0044; Fig. 2B).

#### TGF-β1 and VEGF protein levels

To quantify the expression of known angiogenic factors (TGF-β1 and VEGF) produced by the control and tca cells, cell culture media from both cell types were analyzed by ELISA. The mean level of secreted TGF-β1 was significantly higher in tca cells than in control cells (tca: 173.8±13.0 pg/mL; control: 59.9±4.5 pg/mL; *P = *0.0012; Fig. 2C). Similarly, the mean level of secreted VEGF protein was significantly higher in the supernatant of tca cells than in control cells (tca: 1742.6±14.1 pg/mL; control: 953.2±23.8 pg/mL; *P* = 0.0001; Fig. 2D).

#### HUVEC migration assay

**Figure 3:**
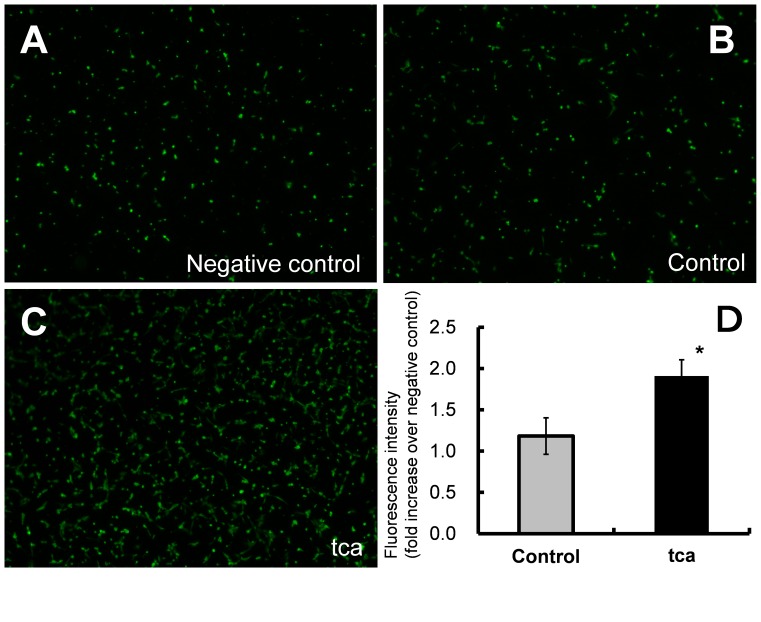
HUVEC migration using ESCC-DR culture supernatants. Culture supernatants were collected from control cells incubated in the medium without TCA and tca cells incubated in the medium with 2 mM TCA for 24 h. (A–C) Fluorescence microscopy was used to detect calcein-labeled HUVEC cells migrating through fibronectin-coated membranes toward a negative control solution (HBSS) (A), control cell culture supernatant (B) and tca cell culture supernatant (C). (×40) (D) Comparison of HUVEC migration between culture supernatant of tca vs. control cells (*P<0.05). The fluorescence intensities of control and tca supernatants were normalized to that of the negative control.

Then, we examined HUVEC migration using supernatants collected from the control and tca cells. Fluorescence microscopy demonstrated that HUVECs migrated through fibronectin-coated membranes toward the negative control solution (HBSS; Fig. 3A) or supernatants of the control (Fig. 3B) or tca cells (Fig. 3C). The rate of migration in tca cells was 1.62-fold higher than that in control cells (tca: 1.91±0.20-fold, control: 1.18±0.22-fold, *P* = 0.0348; Fig. 3D).

### Effects of TCA Treatment on Cell Invasion *in vitro*

#### Cell morphology

**Figure 4:**
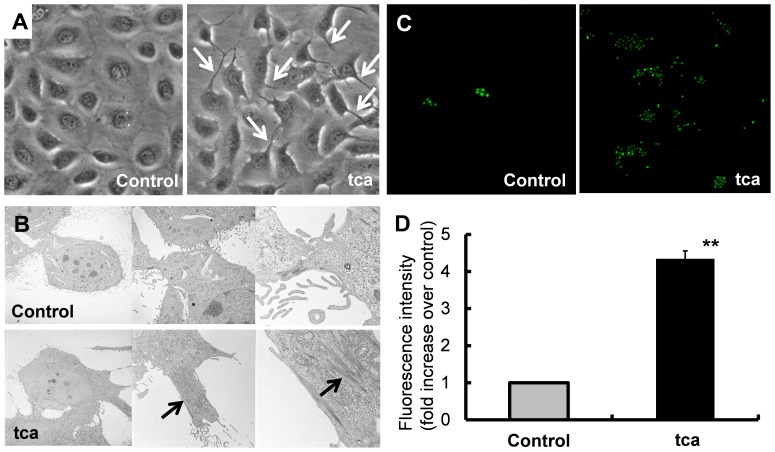
Effects of TCA exposure on ESCC-DR cell morphology and invasion. (A) Podia formation (white arrow) in tca cells (magnification×400). (B) Thick bundles of actin filaments (black arrows) associated with podia formation (magnification×1740, ×4860 and ×18400). (C) Fluorescence microscopy demonstrated the invasion of control and tca cells through Matrigel-coated membranes toward DMEM containing 5% FBS. (D) The rate of invasion was four-fold higher in tca cells compared with control cells (**P<0.01).

There were no remarkable morphological changes for approximately 1 month after the administration of TCA. One month after TCA exposure, thick cell projections (podia) were observed in tca cells (Fig. 4A), with the number of podia increasing with time. We used electron microscopy to study the fine structure of each cell projection. Many short and thin projections without thick actin bundles were seen in control cells. On the other hand, thick bundles of actin filaments were observed in tca cell projections (Fig. 4B). The projections filled with thick actin filaments were morphological similar to filopodia.

#### Cell invasion

Invasion assays were performed in Matrigel-coated Transwell chambers. The number of invading cells was measured after 48 h. Fluorescence microscopy images showed that the control and tca cells (Fig. 4C) had passed the Matrigel-coated membranes to reach the 5% FBS/DMEM solution. The rate of invasion for tca cells was 4.34±0.21-fold higher than that for control cells (*P* = 0.0005; Fig. 4D).

### Effects of TCA Pretreatment on *in vivo* Tumor Growth

#### Xenograft tumor growth

**Figure 5:**
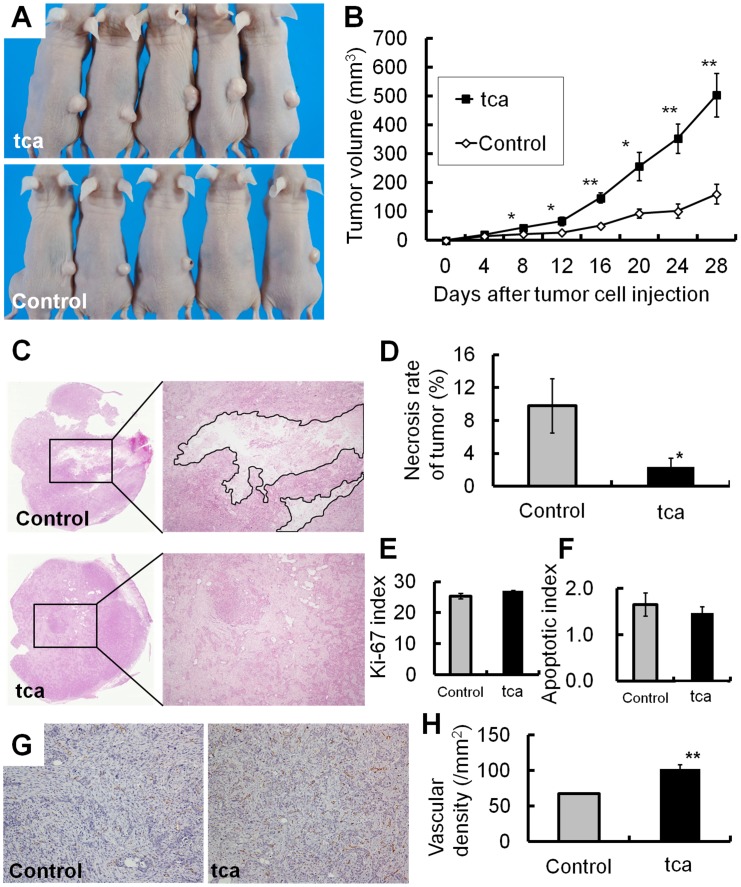
Effects of TCA exposure on tumor xenografts. (A) Tumor xenografts derived from tca and control cells after 28 days. (B) Growth curves of tumor xenografts from tca and control cells. TCA pretreatment significantly enhanced *in vivo* growth at day 28 after tumor cell injection (*P<0.05, **P<0.01). (C) Necrosis (marked with black rim) in tumor xenografts from tca and control cells (magnification×40). (D) Necrosis was significantly reduced in tca cells compared with control cells (*P<0.05). (E, F) There was no significant difference in the mean Ki-67 index (E: *P = *0.0746) or mean apoptosis index (F: *P = *0.5151) between tca and control cells. (G) Immunohistochemical detection of CD31-positive vascular endothelial cells (magnification×200). (H) Vascular density was significantly higher in tca cells than in control cells (**P<0.01).

To evaluate the effects of TCA exposure on cancer progression *in vivo*, both control and tca cells were subcutaneously injected into nude mice. TCA exposure before the injection induced an appreciable increase in tumor volume on day 28 after cell injection compared with control cells (tca: 504.4±76.0 mm^3^; control: 161.3±33.9 mm^3^; *P* = 0.0033; Fig. 5A, B). No metastases to distant organs were noted.

#### Proportion of tumor necrosis

The proportion of tumor necrosis was compared between tca and control cells (Fig. 5C). The mean proportion of necrosis was significantly lower in tumors from tca cells than in those from control cells (tca: 2.4±1.1%; control: 9.8±3.3%; *P* = 0.0496; Fig. 5D).

#### Indices of Ki-67-positive cells and apoptosis in tumor xenografts

We calculated Ki-67-positive and apoptotic cell indices in tumor xenografts. There was no significant difference in the Ki-67 index (tca: 27.1±0.16%; control: 25.4±0.88%; *P = *0.075; Fig. 5E) or in the apoptotic index (tca: 1.47±0.13%; control: 1.65±0.25%; *P = *0.52; Fig. 5F) between tca and control cells.

#### Vascular density in the tumor xenografts

To determine why the proportion of necrosis was significantly lower in the tumors derived from tca cells, we examined the influence of tumor angiogenesis on cancer progression. The density of CD31-positive vasculature was higher in tumors from tca cells compared with those from control cells (tca: 102.3±6.2/mm^2^; control: 67.9±0.8/mm^2^; *P* = 0.0018; Fig. 5G, H). This result indicates that TCA exposure promotes tumor angiogenesis.

## Discussion

We successfully demonstrated that continuous TCA exposure induces ESCC tumor progression. This phenomenon cannot be explained exclusively by the influence of TCA on DNA damage or carcinogenesis-related pathways because TCA exhibits neither mutagenicity nor genotoxicity [31], [32]. Continuous TCA exposure affects cancer progression directly and indirectly. According to the present data, TCA directly enhances invasiveness of ESCC-DR cells *in vitro*, which is associated with TGF-β1 release from cancer cells; TCA also indirectly accelerates tumor growth (by reducing cell loss) *in vivo* through the promotion of angiogenesis mediated by the migration of vascular endothelial cells.

Bile acids exert cytotoxic effects by solubilizing polar lipids such as phospholipids and cholesterol, leading to disintegration of the plasma membrane and death of intestinal epithelial cells [33]–[36]. During preliminary experiments, we incubated ESCC-DR cells with a 2 mM solution of cholic acid (CA), DCA, TCA, glycocholic acid (GCA), taurodeoxycholic acid (TDCA), or glucodeoxycholic acid (GDCA). In most cases, this treatment resulted in a level of cell death that precluded maintenance of viable subcultures. Long-term culture was possible only with TCA or GCA (data not shown). Finally, we selected TCA for this study because of its solubility in the stomach, i.e., TCA has pK_a_ of 1.9 and GCA has pK_a_ of 3.8 [37].

Some authors have reported various effects of bile acids not only on cell death but also on cell proliferation. Nishioka et al. reported that CDCA stimulates proliferation of ESCC cells (TE2R, TE3, TE13, and TE15) through a mechanism involving regulation of the G1 phase [38]. In contrast, Zhang et al. showed that 3-day exposure to CA, DCA, TCA, TDCA, taurochenodeoxycholic acid, GDCA, or glycochenodeoxycholic acid (50–500 µM) inhibits growth and induces apoptosis in ESCC cells (Eca109) [39]. These findings suggest that the effects of bile acids on cell growth vary depending on the type of bile acid as well as the cell line under study. In the present work, FACS analysis showed that treatment with 300 µM DCA for only 24 h induced a G1 arrest and a small amount of apoptosis in control cells. In contrast, brief treatment with 2 mM TCA enhanced S-phase entry in both the control and tca cells and did not induce G1 arrest or apoptosis.

We attempted to clarify the mechanism underlying cancer progression induced by continuous TCA exposure and examined the expression of NF-κB, Akt, Erk, and Cox2 in tca and control cells. We could not detect the expression of NF-κB p65 subunit in the ESCC-DR cells (data not shown). Phosphorylation of Akt and Erk were transiently induced by TCA in the control and tca cells. In tca cells, TCA did not enhance the phosphorylation of Erk but the baseline phosphorylation level was higher than that in control cells. In addition, the expression levels of Cox2 in tca cells were higher than those in control cells. These findings suggest that Erk and Cox2 may be involved in cancer progression elicited by TCA.

The *in vitro* and *in vivo* effects of TCA on cell proliferation were different in our experiments. There was no significant difference in the Ki-67 index of tumor xenografts between the TCA pre-treatment and control groups. These findings confirm the notion that reduction in cell loss rather than enhancement in cell proliferation is the rate-limiting factor for tumor growth *in vivo* [40]. Folkman et al. have shown that the growth of solid tumors takes place in 2 stages, the avascular and the vascular stage [41]. In the avascular stage, a tumor remains dormant because of considerable cell loss, whereas in the vascular stage, tumor growth is accelerated by angiogenesis, which allows survival of tumor cells [41]. Experiments on Barrett’s carcinoma cell line suggest that DCA stimulates VEGF expression [42]. Our results also showed that long-term continuous TCA exposure promotes cancer angiogenesis by inducing TGF-β1 and VEGF expression. VEGF-A and TGF-β1 expression were maintained in tca cells cultured in medium without TCA (data not shown). These proangiogenic factors allow tumors to ward off necrosis and thus survive during hypoxic conditions.

Cancer cell migration is mediated by the formation of cellular protrusions containing actin-rich organelles [43]. The most extensively characterized protrusive structures are the lamellipodia, consisting of a dense meshwork of branched or cross-linked actin filaments [44], [45]. Continuous lamellipodia protrusion and ruffling are frequently accompanied by the formation of parallel actin filaments bundles most frequently termed filopodia [46]. Filopodia are involved in adhesion, migration, invasion, survival, and proliferation of cells [47]. In the present study, the cellular projections that appeared in tca cells and contained thick bundles of actin filaments were morphologically similar to filopodia. Moreover, our *in vitro* experiments indicated that the protein and mRNA levels of TGF-β1 released from the ESCC cells were significantly higher in tca cells, and TCA pretreatment induced a 4-fold increase in invasion rate. It has been reported that TGF-β1 stimulates the invasion of lung cancer cells, which is accompanied by marked changes in cell morphology, such as the appearance of numerous long filopodia filled with actin filaments [48], [49]. Thus, this enhanced invasiveness is probably due to the TCA-mediated formation of filopodia induced by TGF-β1 released from ESCC cells. However, we could not detect any metastases to the other organ in this system using nude mice. Further studies are required to investigate the effects of TCA exposure on cancer cell invasion *in vivo*.

In summary, our results suggest that continuous TCA exposure stimulates ESCC tumor progression. This phenomenon is mainly due to a reduction in cell losses, resulting from enhanced vascular development caused by TCA-induced production and release of TGF-β1 and VEGF-A by ESCC cells. In order to develop new strategies for the prevention and treatment of esophageal cancer, it needs to be further investigated whether the exposure to other bile acids has similar effects.
